# The Effect of Self-Referential Expectation on Emotional Face Processing

**DOI:** 10.1371/journal.pone.0155576

**Published:** 2016-05-13

**Authors:** Mel McKendrick, Stephen H. Butler, Madeleine A. Grealy

**Affiliations:** 1 School of Life Sciences, Heriot-Watt University, Edinburgh, United Kingdom; 2 School of Psychological Sciences and Health, University of Strathclyde, Glasgow, United Kingdom; University of California, San Francisco, UNITED STATES

## Abstract

The role of self-relevance has been somewhat neglected in static face processing paradigms but may be important in understanding how emotional faces impact on attention, cognition and affect. The aim of the current study was to investigate the effect of self-relevant primes on processing emotional composite faces. Sentence primes created an expectation of the emotion of the face before sad, happy, neutral or composite face photos were viewed. Eye movements were recorded and subsequent responses measured the cognitive and affective impact of the emotion expressed. Results indicated that primes did not guide attention, but impacted on judgments of valence intensity and self-esteem ratings. Negative self-relevant primes led to the most negative self-esteem ratings, although the effect of the prime was qualified by salient facial features. Self-relevant expectations about the emotion of a face and subsequent attention to a face that is congruent with these expectations strengthened the affective impact of viewing the face.

## Introduction

The aim of the current study was to investigate whether expectations arising from written statements can prime attention to facial features and whether such a processing pattern subsequently impacts on self-esteem. That is, can such written statements provide a context that influences behaviour and subsequent feelings? This is a theoretically important issue which to date has been largely unexplored.

The human face can be argued to be one of the most fundamentally important objects in social interaction. Specific neural locations have been argued to be attributed to faces (e.g. [1,2] although see [3]). Experimental evidence indicates that faces have an advantage in competition for attention (e.g. [4, 5, 6, 7]). Many face processing studies have not adequately explored the nature of the context in which faces are perceived and judged. Whilst important for our understanding of emotional face processing per se, it additionally has potential implications for theories relating to the development and maintenance of social anxiety.

Our interpretation of human emotion is augmented, directed and in some cases confused by powerful social and environmental cues relating to an individual’s gaze, body posture, language and situation, and in some cases by purely endogenous processes. With regard to gaze, Adams & Kleck [8] provide compelling evidence of its influence on decision speed to correctly assign emotional labels to faces, with anger and joy more quickly identified from faces with direct gaze relative to averted gaze, and the opposite being the case for the emotions of fear and sadness [9]. Nelson, Adams & Stevenson [10] further developed these findings and reported that the looming effect of an approaching angry face toward the viewer appears to facilitate the accurate identification of facial anger, and also increases the likelihood of an ambiguous angry/ fear blend being labeled as angry, with a similar trend of facilitation being observed for apparent motion *away* from the viewer with fear faces.

Further contextual manipulations of differing natures have been demonstrated to influence emotional judgments, such as the type of emotion portrayed by a preceding face or the nature of the contextual information provided to the viewer. Russell and Fehr [11] reported that the nature of emotional information reported by an observer to be displayed by a face can be directly influenced by the emotion seen by the observer on a preceding face acting as a prime in experimentally predictable ways. More recently, Nhi & Isaacowitz [12] have extended this work into other domains of context, reporting evidence that target facial expression identification can be influenced by pictorial scene context (such as a scene designed to elicit anger) more so than context set by another face. The relationship between context and facial emotion identification was further illustrated by Feldman Barrett & Kensinger [13], who asked participants to make either approach-avoid decisions or to emotionally label faces shown superimposed in neutral contexts. Analysing performance across both types of decision, their results indicated better recognition memory for neutral contextual background scenes in the labelling condition than in the approach-avoid condition when the scene was paired with an emotional face. Such an effect was not observed with objects.

Considerable research has now illuminated how body language acts on facial emotion interpretation. Aviezer, Bentin, Dudarev & Hassin [14] reported that facial emotion recognition can be influenced by body context, with the effect strongest when a face showing disgust was presented upon a body displaying anger or vice versa. However, the manipulation was noted to diminish in effectiveness with other juxtapositions of facial emotion and body contexts (see also [15, 16,17]).

Other contextual manipulations, such as priming information regarding the situation of the model have been demonstrated to influence emotional judgments. Carroll & Russell [18] provided compelling evidence that a verbally described situation has a strong influence on the emotion an observer will assign to an emotional face, rather than being purely driven by the emotion signaled by the face itself, when the verbal situation is plausibly consistent with the facial emotion. More recently, Suess, Rabovsky & Rahman [19] argue that even novel experimentally acquired socially relevant negative biographical information about a stranger effects the processing of neutral facial expressions, resulting in the neutral facial expression being classified as more negative in valence than a neutral face associated with more neutral information about the person.

Recent research has examined the influence of the *personal* relevance of another’s emotional state. Herbert, Sfärlea, and Blumenthal [20] found that personal relevance of emotional words influenced semantic processing stages when viewing fearful, happy and angry faces, using contextual cues with personal and non-personal affective/non-affective labels such as ‘my panic’ or ‘her anger’. The researchers suggest that the processing of the emotional content of word pairings may have involved a top-down influence on the processing stages of facial features processing by way of anticipation of the emotion of the upcoming face stimulus. Complementary imaging evidence supports the view that attributing the cause of the emotion shown to the observer influences neural processing of the face. Boll, Gamer, Kalisch & Buechel [21] demonstrate that personal context, manipulated by the degree of scenario driven personal relevance of an emotive face, influences amygdala activity in the perceiver in a pattern in line with previous findings with gaze cues in such emotional faces, such that angry faces whose cause was attributed directly to the perceiver elicited higher amygdala activity than angry faces whose cause was attributed to another person.

Attention is known to play an important role in the generation of voluntary saccadic eye movements. When we move our eyes to a location, through the generation of a voluntary saccade, we tend to move our attention there too [22], indeed the linkages between attention and eye movements have been thoroughly explored (for an excellent overview see [23]). The study of eye movements has been particularly illuminating regarding how our eyes are attracted to faces [24, 25], and has illustrated differential eye movement patterns when judging facial emotion [26].

Importantly, for the purpose of the present study, emotional cues from body language have also been shown to influence overt eye movement behaviour. Aviezer et al [15] reported that emotion cues from body posture guide fixations to facial features that were consistent with viewers’ expectations. Finding, for example, that there were a higher proportion of fixations to the eye area compared to the mouth area in angry faces primed by a complementary angry body posture, however when the same face was paired with a disgust posture the relative proportions became more equal. This congruency effect suggests that the expectation of viewing an angry face guides fixations in a pattern that is consistent with viewing angry faces, with the same being the case for the expectation of seeing a disgust face.

Aviezer et al [15] appear to provide clear evidence that contextually priming a facial emotion with either a congruent or incongruent emotive body posture leads to direct influences on eye movement behaviour. These findings are supported by further research. For example, Shields, Engelhardt & Ietswaart [16] also examined eye movements in participants exposed to both emotionally congruent and incongruent face body combinations and reported that, whilst incongruent pairings caused a bias to choose the emotion displayed on the face, it was also evident that emotional face-body incongruency led to more fixations to the face and fewer to the body than congruent trials. Thus an incongruent body appears to lead to differential eye movement behaviour per se relative to congruency of face and bodily emotion.

More recently, Noh & Isaacowitz [17] examined the performance of younger and older adults in an emotion identification task where either angry or disgusted faces were similarly shown in either a neutral (head and shoulders pose), congruent or incongruent context. Crucially, the researchers found evidence of differential eye movement behaviour related to contextual manipulation in both first fixation and fixation duration behavior, finding for example that with angry faces the amount of first fixations to the eye relative to other regions differed depending on context.

Whilst such priming in the form of body language appears able to change behaviour, additionally, prior expectation has been shown to influence emotion identification. For example, Barbalat, Bazargani and Blakemore [27] found greater accuracy and shorter reaction times for categorizing fearful or angry faces when they were instructed to specifically identify the presence of one of these emotions in a set of individually presented facial stimuli. In a live social situation, one could equally consider that such expectations may be primed by memories of facial expressions from previous encounters or from internal thoughts regarding the anticipated emotional response to a social encounter. This may be of particular importance to individuals who manifest maladaptive approaches to social interaction. However, it would appear that to date interventions to reduce social anxiety in live social situations by training attention towards a non-socially threatening face have had mixed success (e.g. [28, 29, 30]).

Whilst Herbert, Sfärlea, and Blumenthal [20] appear to indicate that the personal relevance of another’s emotional state can influence semantic processing of emotional images, and research such as that reported by Aviezer et al [15], Shields et al, [16] and Noh & Isaacowitz [17] indicates that primes in the form of discrepant bodily emotion can influence eye movement behaviour, the effects of context manipulation have additionally been trialed using visual imagery. For example, Baldwin, Granzberg, Pippus and Pritchard [31] found that the visualization of an accepting face was associated with greater social performance satisfaction than the visualization of a critical face. What is not clear from the current literature is whether self-relevant primes in the form of written situational narratives may be capable of demonstrating the congruency effects demonstrated by Aviezer et al [15] by actually directly influencing attentional allocation, as indicated by the influence on fixations to emotional facial features that are congruent with expectations.

Indeed a review by Feldman Barrett, Mesquita & Gendron [32] highlighted the important role of context in influencing perception and attention, arguing that the salience of facial features do not govern these processes without the guiding hand of context. However, they cite evidence from studies using visual and non-visual context without differentiating the mechanistic differences involved in these. Indeed, it could be argued that the context effects found in the Aveizer et al study [15], for example, could be an artifact of salience since the postural cues occupied a greater visual space than the facial expressions. This point was noted by Mobbs, Weiskopf, Lau, Featherstone, Dolan & Frith [33] in their study of the effect of emotional movies on ratings of expression and mental state of facial expressions and BOLD activity in an fMRI study. Whilst results indicated congruency effects, the authors noted that the valenced movies were likely to be more salient than the facial expressions. To investigate the effect of context alone on attention it may be prudent to take it out of the visual domain by using verbal or written context. The fMRI data from the Mobbs et al study suggest a role for the STS in congruency effects. The suggestion is that activity in this area represents the combination of social cues from context and salient features. The STS has also been found to be sensitive to context when processing familiar versus novel faces in an fMRI study by Apps and Tsakiris [34], who suggested that context driven expectations about upcoming stimuli will initiate processing about the expected stimulus prior to stimulus presentation. However, whilst this may provide an explanation for how visual primes guide attention since the STS has been implicated in processing physical aspects of faces and in processing objects, non-visual primes may operate differently.

Congruency clearly influences the neural processing of faces. Diéguez-Risco, Aguado, Albert & Hinojosa [35] employed valenced sentence primes to test congruency effects between context and facial expressions. Results indicated that incongruence was associated with larger N170 amplitudes for happy and angry faces at 155-180ms and larger LLP amplitudes for happy faces (550-700ms) post stimulus presentation. Participants also made more rapid explicit congruency judgements for both happy faces with positively valenced sentences and angry faces with similarly positively valenced sentence primes. This indicates that context in the form of non-visual primes does appear to influence both early and later neutral processing and explicit judgments.

Whilst the question as to whether primes as facilitators of context can influence real social behaviour is of theoretical interest, the question is quite relevant for a number of psychiatric disorders which have been associated with a negativity bias for processing faces, and the mechanisms that drive such a bias. For instance, Major Depressive Disorder (MDD) has been associated with impaired recognition of happy faces and a misinterpretation of neutral faces as sad [36]. MDD has also been associated with an attentional bias towards sad faces [37, 38]It is possible that attention to negative faces is primed by the expectation that a negative face is more likely to be encountered.

Indeed, expectancies of negative outcomes in social situations have been associated with social anxiety [39, 40]Automatically generated anxiety based schemas in social situations create an expectancy of a negative outcome, resulting in some cases in self-fulfilling prophecy. Whilst two common cognitive behavioural models of social anxiety (i.e.[41, 42]) conflict in their predictions regarding attention to facial expressions/gestures, both predict dysfunctional facial attention. Clark and Wells [41] predict that anxiety is maintained in a social situation by decreased attention towards social cues, precipitated by increased self-focused attention. This results in missed opportunities for positive reinforcement from approving audience responses. Rapee and Heimberg [42] however argue that attention is split between imagining ones’ own performance and scanning the audience for signs of social disapproval.

Social anxiety thus may be reinforced or precipitated by a disproportionate lack of attention to positive social cues [41] or an exaggerated allocation of attention to negative social stimuli [42], triggering a negative cycle of attentional, cognitive and behavioral effects that culminate in the maintenance of social anxiety [43]. However, the question of whether expectancies generally affect social attention and perception or whether this is specific to social anxiety has not been fully explored. In light of the findings on congruency effect discussed earlier it may be the case that attentional biases found in social anxiety are simply an extension of a general pattern of attention guided by context.

It is difficult to know how much biased attention towards a negative face may impact on the self-referential cognitions of an individual. It may be the case that more attention paid to a positive or negative face will produce feelings that are consistent with the positive or negative valence of the face. On the other hand, the expectation of feeling positive or negative may lead to greater attention to a consistent positive or negative face. Moreover, this may be magnified with expectations that are self-relevant.

In this light, cognitive reappraisal, which refers to the reinterpretation of information generally during or after presentation, can lead to an increase in positive emotional experience as well as a reduction in negative emotional experience [44]. Therefore, it is possible that biased attention towards a negative face may subsequently impact on how the individual feels and thinks about themselves. It may be the case that if more attention is paid to a negatively valenced face, this will produce a more negative cognitive appraisal that is consistent with the negative valence of the face. Thus, a negative prime (be it internally generated or provided by an experimenter) may lead to greater attention to a negative face, which in turn may make thoughts and feeling about the ‘self’ more negative. Moreover, this may be pronounced with primes which the individual sees as self-relevant. Therefore, the context in which a face is viewed may influence how the viewer personally relates to the face *and therefore may affect processing* at several stages including the way in which the face is attended to, categorized and responded to at an affective level.

This proposal is supported by studies of optimists that have revealed that rather than the valence of information being presented to them being the most important factor in engaging attention, it is the degree to which the information was relevant to self that influenced attention and recall [45, 46]. The degree of self-relevance when processing emotional faces may similarly influence the way in which they are encoded and processed.

The purpose of the present study is to examine whether language primes as context for an emotional face processing task would be capable of guiding attention, as indexed by overt eye-movements, to facial emotion cues. If, as Herbert et al [20] have suggested, valence cues generate anticipatory neural activity then it may lead to visual attention being drawn to facial emotion cues that are consistent with that particular expectation. Generally in terms of face processing, the eyes receive the predominance of attention, possibly due to the degree of rich information that can be elicited from eyes [47]. If it is the rich social information that can be derived from the eye region that draws greater attention, then this may be enhanced by information provided beforehand by the way of a self-referential prime, which may also subsequently influence the personal impact of the engagement, creating a personal context. Can language primes as context for emotional face processing be capable of guiding attention to facial emotion cues? To date we would argue that such a potential influence on our attention to faces and its subsequent impact has not been adequately addressed in face processing research. The present study will directly assess the impact of negative and positive self-referential primes contrasted with primes that lack self-referential value.

We know, from a salience driven perspective (e.g. [48]) that different emotions are routinely categorized from different parts of the face, with attention being guided by the salience of features (whites of eyes and size of mouth). Additionally, Calder, Young, Keane and Dean [49] have demonstrated that anger, fear and sadness appeared to be processed on the basis of the upper face, whilst happiness and surprise were categorized on the basis of the bottom half.

However, Malcolm, Lanyon, Fugard and Barton [50] have also shown that task demands can guide eye movements, suggesting that attention is not directly coupled with fixations, which they argued are directed by top-down strategies in addition to perceptual processes. Information encoded between fixations could influence the location of the subsequent eye fixation, and this may be driven by task effects and contextual information. Therefore, expectations may prime the perception and potentially the attentional processing of a face.

In order to evaluate the effect of context in the present study, the effect of positive, negative and neutral contextual linguistic prime information that was either directed towards or away from the perceiver was directly investigated. Several studies have used composite faces with different emotions being presented on each side of the face (i.e. [51, 52]), but in order to investigate the effects of participants attention to the eyes or mouth, we split the faces horizontally to composite images with oppositely valenced expressions on the top and bottom half of the face (similar to Calder et al [49]). This manipulation afforded us the opportunity to directly examine the influence of primed expectations relating to the face on attentional allocation to features compatible with that expectation in otherwise ambiguous faces. Schubo, Guido, Meinecke & Abele [53] found in a visual search paradigm using schematic faces that facial features when detected faster than whole faces in a masked condition, suggesting that facial features in themselves can be more salient than whole faces when the task difficulty is increased. In this case the inclusion of composite faces increases the ambiguity of the face and conflicting features have to compete for salience, although the composite faces used in the current study are whole faces rather than the disembodied schematic features used the Shubo et al study.

The aim of the current study, employing eyetracking as a measure of visual attention, was thus to investigate whether expectations arising from written statements of a positive, negative or neutral context primed attention to facial features in faces displaying both congruent features (e.g. happy eyes and happy mouth) and faces with incongruent features (e.g. happy eyes and sad mouths), and whether this processing pattern subsequently impacted on levels of self-esteem.

Specifically three hypotheses were made.

The first, in line with previous findings was that a higher proportion of fixations and longer dwell time would be made to the eyes in general. This hypothesis is principally viewed as ‘housekeeping’ that our stimuli have been approached by our participants in line with previous findings in the literature (e.g. [47, 54]). The second and third hypotheses are of richer theoretical importance.

The second hypothesis was aimed at investigating the possibility that primed expectations relating to the cause of the facial expression to be viewed would guide participants’ attention to features that were compatible with this expectation. In line with Aviezer et al [15], it was hypothesized that with *incongruent* composite faces (i.e. happy eyes and sad mouth or vice versa), the primed expectation would guide fixations towards the feature congruent with the expectation (i.e. sad eyes when cued to expect a negative face and happy mouths when cued to expect a positive face).

The third hypothesis was in line with the Rapee and Heimberg’s model of social anxiety [42] which postulates that attention to negative external social cues is integrated with negative self-focus. That is the categorization of the face and the impact on self-esteem would be intensified (indicated by more extreme ratings) when the person was given a congruent self-referential prime. Therefore, it was predicted that negative self-referential prime information would have a greater impact when it coincides with attention to a negatively perceived face.

## Method

### Participants

Nineteen participants (3 male, 16 female, aged 18 to 54; *M*_*age*_ = 30.0 years) were recruited from a community sample to participate. Inclusion criteria included English speakers with normal or corrected-to-normal vision, free from visual deficits. Exclusion criteria included a history of substance abuse for past two years; a current or recent psychiatric disorder or neurological illness. All participants provided written consent, by means of an approved consent form to take part in the study. Ethical approval for the study was obtained from the Ethics Committee of the School of Psychological Sciences and Health, University of Strathclyde.

### Apparatus

Participants were seated in a dimly light room, 57 cm from the monitor and wore a lightweight headset comprising a head camera and two eye cameras placed just below the eyes. Eye-movements were recorded using the SR Research Ltd. Eyelink II system (SR Research Ltd., Mississauga, Canada) using pupil center at 500Hz and .01° spatial resolution. Fixations were defined as the intervals in-between saccades during which the eyes are relatively static. Saccades and fixations were defined using the SR Research saccade detection system, with a combined threshold of minimum saccade velocity (>30°/s), acceleration (>8000°/s) and amplitude (>.15°). The proportion of fixations referred to the percentage of total fixations in a trial falling within the area of interest. The proportion of dwell time referred to the percentage of trial dwell time spent on the area of interest.

Stimuli were presented centrally on a ViewSonic G90ft 19 inch color monitor attached to a Phillips personal computer controlled by Experiment Builder software (SR Research Ltd., Mississauga, Canada).

### Stimuli

Thirty whole photo face stimuli: five male (models 21M; 22M; 23M; 24M; 28M) and five female models (01F; 02F; 3F; 05F; 06F) consisting of three emotional sad, happy and neutral expressions were selected from the NimStim Set of Facial Expressions [55]. These were then manipulated into five combinations of composite face types.

Composite faces were constructed using Photoshop Elements 5 (Adobe Systems Incorporated, San Jose, USA) by combining various arrangements of happy or sad upper face portions from the eye area (defined as above the bridge of the nose); lower face portions from the mouth down (from the tip of the nose down) with neutral mid sections (including the nose). This gave rise to five different emotional configurations for each of the ten models; happy eyes and mouth (happy), happy eyes and sad mouth (happy/sad), sad eyes and happy mouth (sad/happy), sad eyes and mouth (sad) and neutral eyes and mouth (neutral). Each facial image, matched for skin color and differences in hair, was framed with a standardized oval so that when they were viewed the vertical visual angle was 17.2° and the horizontal 13.9°.

Each of the 50 stimuli was then paired with five primes (positive self-referential, positive non-self-referential, negative self-referential, negative non-self-referential and neutral). This resulted in 250 trials which were presented in five randomized blocks with each block containing 50 trials.

A final total of 250 prime statements were employed in the study, to allow for a strong factorial balance across face types and self or other prime relevance. Initially, 250 prime statements were constructed (and piloted) to be positive, negative or neutral and self or non-self-referent with only the target words changed in valenced statements so that each was of equal length and structure. Sentences were manipulated to include subject pronouns that were either personally self-relevant (e.g. ‘you’) or non-self-relevant (e.g. ‘they’), with either a positive or negative noun, e.g. ‘Kate was convinced that with [you] *or* [them] the night would be a [disaster] *or* [success]’. Thus, the valence of each emotional statement depended on a depressogenic word or its antonym such as ‘crestfallen’ or ‘elated’. For example, ‘Ben gladly approached you to join him’ (positive self-referential); ‘Ben gladly approached them to join him’ (positive non-self-referential); ‘Ben reluctantly approached you to join him’ (negative self-referential); ‘Ben reluctantly approached them to join him’ (negative non-self-referential); ‘Ben opened the window’ (neutral). Neutral sentences were not necessarily the same length and structure as they could not contain reference to self or others without being a social prime.

Statements were rated for valence by 20 independent raters. In order to retain consistency in valence ratings between primes, statements which fell within the range of above 3.5 to 4.5 on a 5 point agreement Likert scale indicating that the statement was positive and between -3.5 to -4.5 for negative, were included in the final selection. Those outside this range were replaced with new statements which were again rated until a final sample of 250 statements was obtained. Neutral statements were accepted if they fell within the range of -.5 to .5. The final selection of statements were then rerated by a separate 27 raters who were asked to rate the valence of each statement on an 11-point scale ranging from –5 (very negative) to 0 (neutral) to +5 (very positive) (e.g. [56]).

A one-way ANOVA on these ratings with prime type (5) as a repeated measures factor revealed a highly significant main effect of prime type, *F*(1.11, 28.79) = 257, *MSE* = 2.45, *p* < .001, *ηp²* = .91 *Power* = 1). Bonferroni contrasts revealed that positive primes (Positive non-self-referential: M = 2.49, SE = .16; Positive self-referential: M = 2.7, SE = .14) were rated significantly more positively than neutral (M = .17, SE = .06, *p* < .001) or negative primes (Negative non-self-referential: M = -2.34, SE = .16; *p* < .001; Negative self-referential: M = -2.62, SE = .18). The negative primes were rated significantly more negatively than neutral primes (*p* < .001). Furthermore, positive self-referential statements were rated significantly more positively than positive non-self-referential statements (*p* < .001), and negative self-referential statements were rated significantly more negatively than negative non-self-referential statements (*p* = .001).

Although the whole face images were selected from a pre rated database, composite faces were not rated in the absence of primes in a bid to control for individual differences during the rating process. When tasked with rating an ambiguous face people are unlikely to do this without an accompanying internal narrative and thus the ambiguity may increase processing time, potentially shifting a relatively automatic process to a more controlled decision making process. Thus composite faces may be more sensitive to the effects of individual differences. Controlling the internal narrative through neutral prime statements, may allow for a more standardized baseline to test the effect of valenced self-referential and non-self-referential primes.

### Procedure

**Fig 1:**
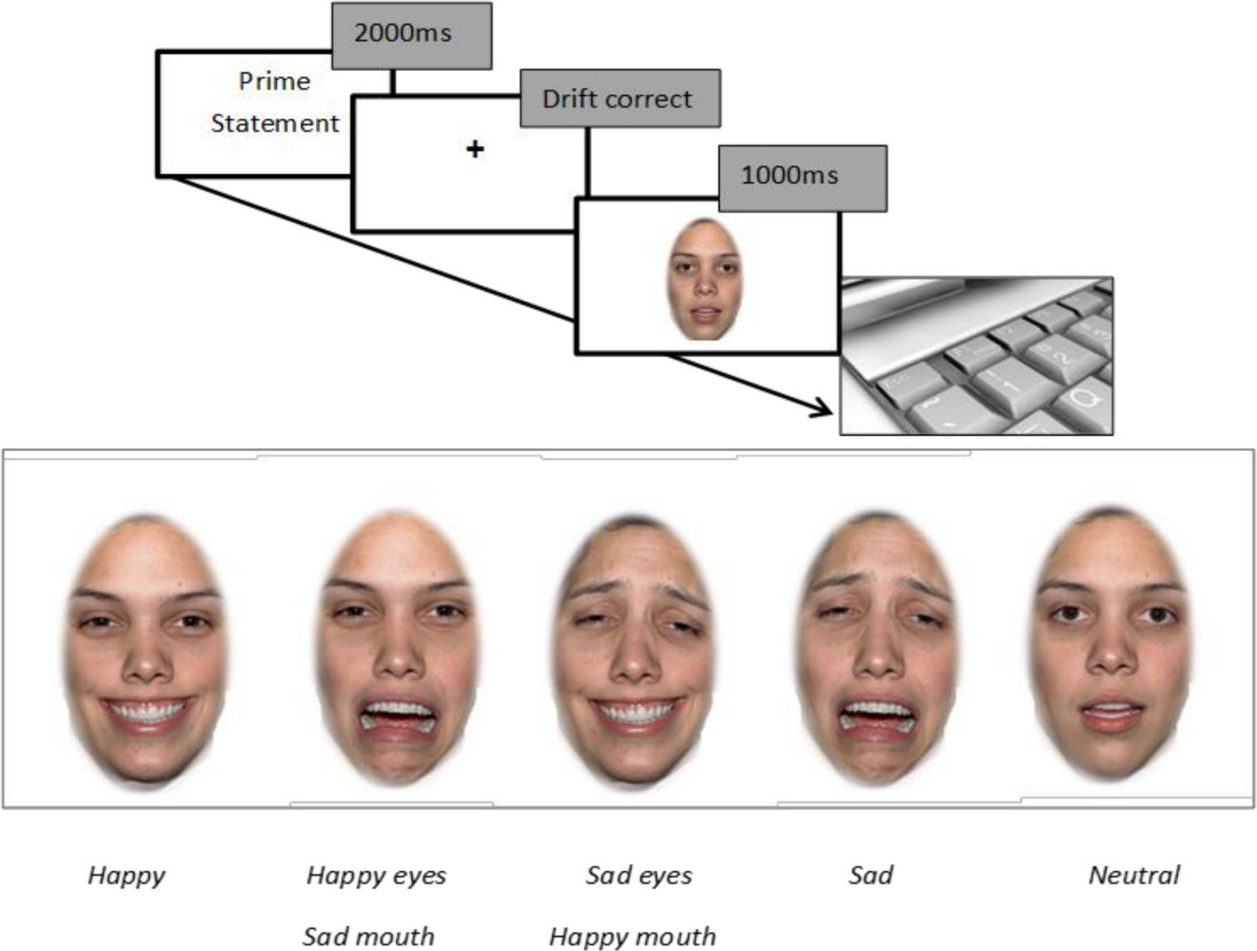
Schematic of experiment.

At the beginning of each trial, the participant was presented with a prime for 2000 ms in the form of a statement about the person whose face was subsequently presented on the screen. After each prime, participants were presented with a drift correction central fixation dot, followed by a face for 1000 ms which participants were instructed to look freely at (Task 1). After each face disappeared from the screen participants were asked to rate the valence of the emotion of the face that they had just been looking at via a keyboard response with options from ‘extremely positive’ (rated 1); ‘slightly positive’; ‘neutral’; ‘slightly negative’; to ‘extremely negative’ (rated 5) (Task 2). Participants were then asked to indicate how being confronted with this face made them feel about themselves by indicating a response from ‘extremely positive’(rated 1); ‘slightly positive’; ‘neutral’; ‘slightly negative’; to ‘extremely negative’ (rated 5) (Task 3). The experimental sequence is illustrated in Fig 1. Participants were asked to complete a set of 12 practice trials before the experimental procedure commenced and they were debriefed after the end of the experiment. Repeated measures ANOVAs were used to analyze the eye movement data when viewing the faces and the subsequent ratings, and Greenhouse Geisser corrections were applied where appropriate.

## Task 1: Freeview Eye-Tracking

It was predicted that the eyes would receive a higher proportion of fixations and dwell time than mouths. Moreover, it was expected that happy mouths would be more salient than sad mouths and sad eyes would be more salient than happy eyes, again indicated by a higher proportion of fixations and dwell time.

Crucially, it was hypothesized that with incongruent composite faces (i.e. happy eyes and sad mouth or vice versa), the nature of prime statements would guide fixations towards the facial feature that was congruent with the prime. Specifically, it was predicted that when there was a negative prime there would be a higher proportion of fixations and dwell times to facial features that would display negative emotions, and similarly for a positive prime there would be a higher proportion of fixations and dwell times to facial features that would display positive emotions. Additionally, it was expected that this congruence effect would be intensified with a self-referential prime.

### Results

Trials where the first saccade was initiated less than 80 ms after face stimuli onset, or where the initial fixation was more than 1° from the central fixation point were excluded. This resulted in a total of 3.8% of trials being discarded (an average of 9.5 trials per participant). Fixations and dwell time were analyzed as a mean proportion to areas of interest relative to the number of valid trials in relation to the eye area (defined as the area on the face above the bridge of the nose); lower face portions from the mouth down (defined as the area on the face from the tip of the nose down) and neutral mid sections (defined as the area on the face between these two regions). The number of fixations and dwell time within each of the facial areas were then calculated and expressed as proportions of the total number of fixations and dwell time respectively.

### Proportion of fixations

**Fig 2:**
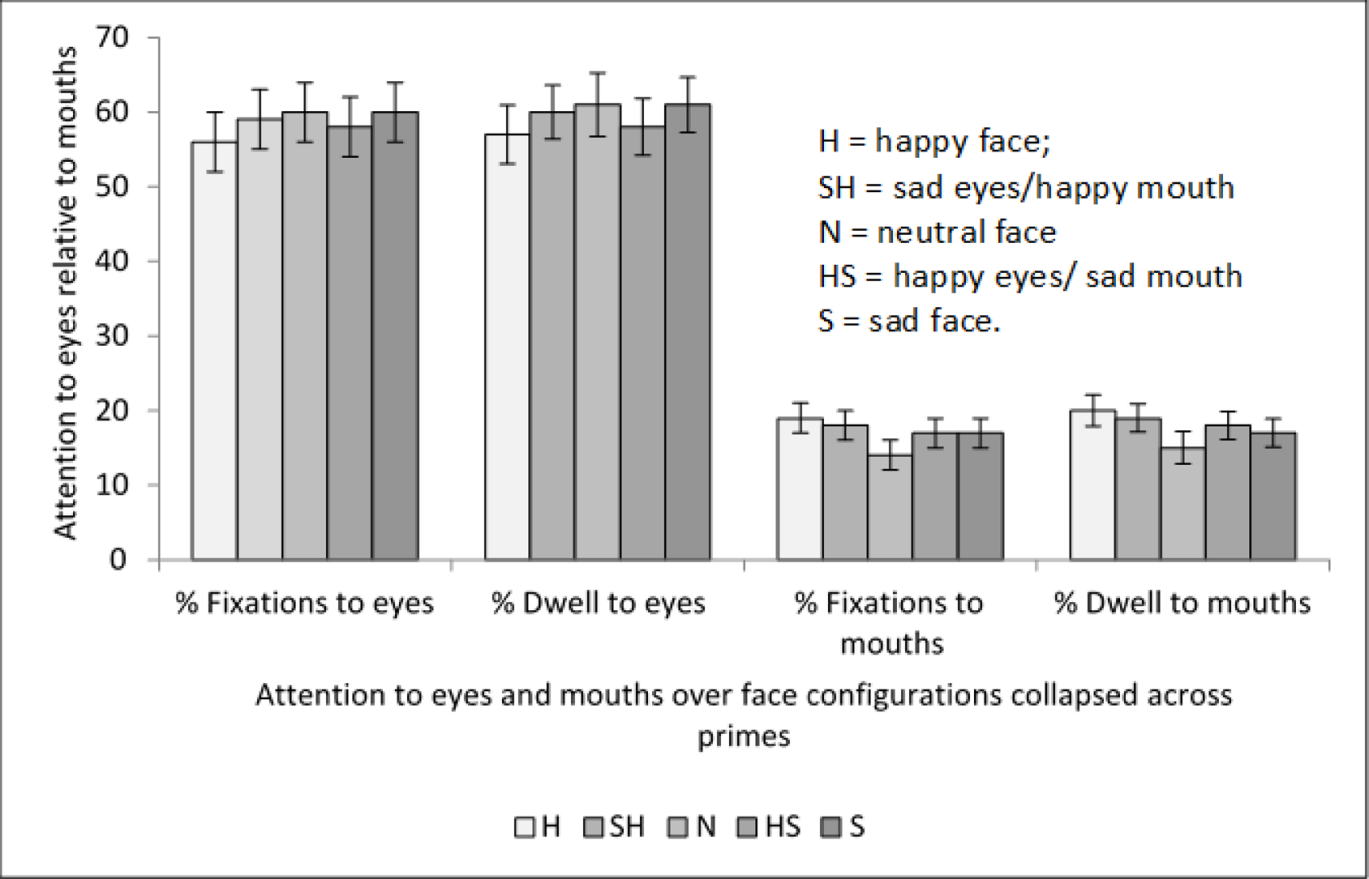
Means and standard errors for percentage of fixations and dwell time to eyes and mouths collapsed across primes.

The mean percentage of fixations to the eyes and mouths are shown in Fig 2. An area of face (3) x prime (5) x emotional configuration (5) repeated measures ANOVA was conducted on the proportion of fixations. There was no significant main effect of prime, *F* (4, 72) = 1.38, *MSE* = .001, *p* = .25, *η*_*p*_*²* = .07. However, as expected, there was a highly significant main effect of the area of face that was fixated, *F* (2, 36) = 57.01, *MSE* = .52 *p* < .001, *η*_*p*_*²* = .76. Bonferroni pairwise contrasts indicated that eyes received a significantly higher proportion of fixations (*M* = .59, *SE* = .37) than mouths (*M* = .17, *SE* = .02, *p* < .001) or noses (*M* = .14, *SE* = .02, *p* < .001). The main effect of emotional configuration was not analyzed since the data were expressed as proportions, that is for each emotional configuration the overall proportion for fixations was always 1.0.

There was a significant area of face x emotional configuration interaction, *F* (4.28, 76.96) = 5.87, *MSE* = .06, *p* < .001, *η*_*p*_*²* = .25, and Bonferroni corrected contrasts indicated that eyes received a significantly lower proportion of fixations in happy eyes/sad mouth faces (*M* = .58, *SE* = .04) than sad faces (*M* = .6, *SE* = .04, *p* = .03) or neutral faces with the difference approaching significance (*M* = .6, *SE* = .04, *p* = .06).

Mouths received a significantly higher proportion of fixations for happy faces (*M* = .19, *SE* = .02) than happy eyes/sad mouth faces (*M* = .17, *SE* = .02, *p* = .01); neutral faces (*M* = .14, *SE* = .02, *p* < .001) or sad faces (*M* = .16, *SE* = .02, *p* < .001). Sad eyes/happy mouth faces (*M* = .18, *SE* = .02) also had a significantly higher proportion of fixations to the mouth than neutral faces (*p* = .01) or sad faces (*p* = .02), but did not differ from happy faces (*p* > .95). This suggests that happy mouths were more salient than others. There was no difference in proportion of fixations to mouths between happy eyes/sad mouth faces or sad faces (*p* = 1). However, the finding that there was no difference in fixation to mouths between happy eyes/ sad mouth faces and sad eyes/happy mouth faces (*p* = .2) suggests that the salience that normally guides fixations to the happy mouth were offset by the sad eyes. There were no other significant differences across the emotional configurations for proportion of fixation to mouths (*p* > .17).

The area of face x prime interaction was non-significant *F* (4.25, 76.3) = 2.38, *MSE* = .01, *p* = .06, *η*_*p*_*²* = .18), as was the prime by emotional configuration interaction, *F* (6.33, 113.93) = .75, *MSE* = .01, *p* = .62, *η*_*p*_*²* = .04). The prime x area of face x emotional configuration interaction was also non-significant, *F*(10.3, 185.49) = 1.05, *MSE* = .01, *p* = .39, *η*_*p*_*²* = .06). These results did not support the hypothesis that context in the form of expectations made through prime statements would guide fixations.

### Proportion of dwell time

Dwell time data essentially followed the same pattern as the fixation proportion data. There was a significant main effect of area of the face, *F*(2,36) = 57.1, *MSE* = .55 *p* < .001, *η*_*p*_*²* = .76. Bonferroni pairwise contrasts indicated that eyes received a significantly higher proportion of dwell time (*M* = .60, *SE* = .38) than mouths (*M* = .18, *SE* = .02, *p* < .001) or noses (*M* = .13, *SE* = .24, *p* < .001). Therefore as predicted, and in line with standard face processing patterns, the eyes received significantly more attention than any other feature.

There was a significant area of face x emotional configuration interaction for proportion of dwell time, *F*(4.07,73.18) = 4.86, *MSE* = .02, *p* < .001, *η*_*p*_*²* = .21. Bonferroni corrected contrasts indicated that eyes received a significantly lower proportion of dwell time for happy eyes/sad mouth faces (*M* = .58, *SE* = .04) than sad faces (*M* = .61, *SE* = .04, *p* = .02). There were no other significant differences across configurations for proportion of dwell time to eyes (*p* > .78).

Mouths received a significantly higher proportion of dwell time for happy faces (*M* = .20, *SE* = .02) than happy eyes/sad mouth faces (*M* = .18, *SE* = .02, *p* = .02), neutral (*M* = .15, *SE* = .02, *p* = .001) or sad faces (*M* = .17, *SE* = .02, *p* < .001). This reinforced the finding that happy mouths were more salient than others. However, there was no difference between dwell times for happy eyes/sad mouth faces (*M* = .19, *SE* = .02) and happy faces (*M* = .2, *SE* = .02, *p* = .66). There was also no significant difference for dwell time to the mouth between happy eyes /sad mouth face and sad eyes/ happy mouth faces (*p* = .26) which again suggests competition between salient features. There were no other significant differences across configurations for proportion of dwell time to mouths (*p* > .12). Means and standard errors are illustrated as percentages of fixations and dwell time to eyes and mouths across emotional configurations in Fig 2.

In tandem with the fixation proportion data, there was no significant main effect of prime for proportion of dwell time, *F*(4,72) = 1.49, *MSE* = .001 p = .08, *η*_*p*_*²* = .07. The area of face x prime interaction was significant *F*(4.76, 85.75) = 2.76, *MSE* = .02, *p* = .03, *η*_*p*_*²* = .13, but Bonferroni post hoc contrasts indicated that this was only in respect of a higher proportion of dwell time to noses in faces preceded by a negative non-self-referential prime than a positive self-referential prime. The prime x configuration interaction was non-significant, *F*(6.48, 116.64) = .48, *MSE* = .01, *p* = .83, *η*_*p*_*²* = .03. The area of face x emotional configuration interaction was also non-significant, *F*(10.09, 181.7) = 1.02, *MSE* = .01, *p* = .43, *η*_*p*_*²* = .05.

As predicted, eye movement results suggested that eyes elicited more attention than mouths and also as predicted, sad eyes were more salient than happy eyes when mouths are sad but not when mouths are happy. This is likely to be due to the influence of the more salient happy mouth which reduced the attention drawn to the eye. Also as predicted, happy mouths appeared to be more salient than sad mouths. However, in the absence of any effects of the prime statements, the central hypothesis that prior expectation regarding the cause of the facial expression would guide participants’ attention to congruent facial features could not be supported.

## Task 2: Valence Rating of Facial Stimuli

After viewing the prime statement and face, in each trial participants were then asked to rate the valence of the emotion of the face that they had just looked at. It was hypothesized that valence ratings would be intensified with congruent self-referential expectations that the expression on the face has been caused by the viewer.

### Results

**Fig 3:**
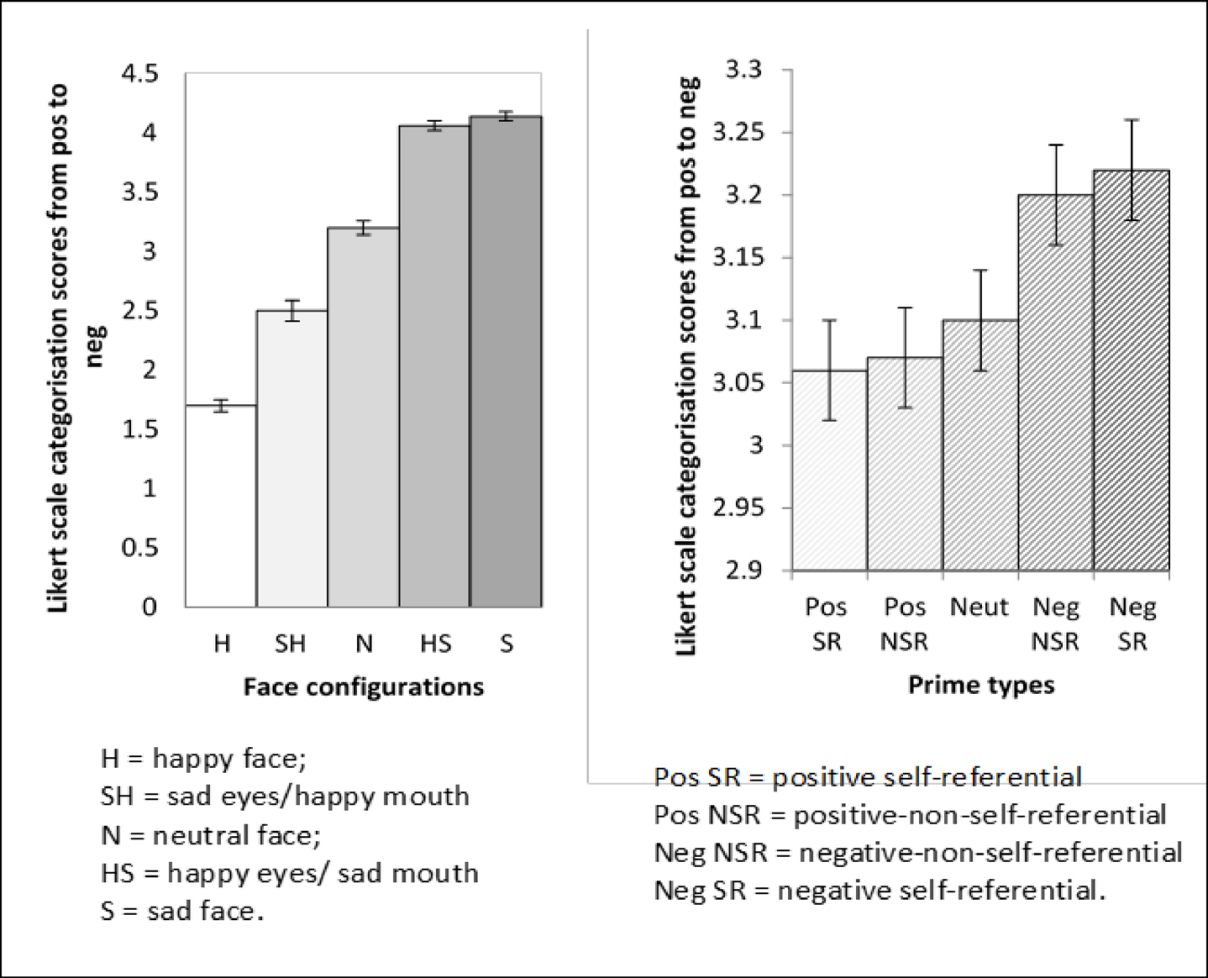
Means and standard errors for valence ratings across emotional configurations collapsed across prime types and for prime types collapsed across emotional configurations.

Means and standard errors for valence ratings across configuration and prime are illustrated in Fig 3. A prime (5) x emotional configuration (5) repeated measures ANOVA was conducted on the valence data. There was a significant main effect of prime, *F*(4,72) = 12.28, *MSE* = .43 *p* < .001, *η*_*p*_*²* = .41. Faces were rated significantly more negatively with negative non-self-referential primes (*M* = 3.2, *SE* = .04) compared to neutral primes (*M* = 3.1, *SE* = .04, *p* = .004) or positive non-self-referential primes (*M* = 3.07, *SE* = .04, *p* = .002). Similarly, faces were rated significantly more negatively with negative self-referential primes (*M* = 3.22, *SE* = .04) compared to neutral (*p* = .01) or positive self-referential primes (*M* = 3.06, *SE* = .03, *p* = .003). However, there were no significant differences in ratings between positive self-referential (*M* = 3.06, *SE* = .03,) and positive non-self-referential (*M* = 3.07, *SE* = .04, *p* > .95) or neutral primes (*M* = 3.1, *SE* = .04, *p* > .95). Furthermore, there was no significant difference in valence ratings of faces preceded by negative self-referential (*M* = 3.22, *SE* = .04) compared to negative non-self-referential primes (*M* = 3.20, *SE* = .04, *p* = .42).

The main effect of emotional configuration was also significant, *F*(3,50) = 332.64, *MSE* = .45 *p* < .001, *η*_*p*_*²* = .95. Bonferroni contrasts indicated that valence categorization was made on the basis of the mouth. Bonferroni corrected contrasts revealed that happy faces were rated significantly more positively (*M* = 1.7, *SE* = .05, *p* < .001) than faces with sad eyes/happy mouths (*M* = 2.5, *SE* = .09, *p* < .001) and happy eyes/sad mouth faces (*M* = 4.06, *SE* = .04, *p* < .001). Furthermore, happy eye/sad mouth faces were rated significantly less negatively than sad faces (*p* = .04). However, despite no differences in eye movements, happy eye/sad mouth faces were rated negatively and were significantly more negatively rated than sad eye/happy mouth faces (*p* < .001). The prime x emotional configuration interaction was not significant, *F*(16, 288) = 1.37, *MSE* = .006, *p* = .16, *η*_*p*_*²* = .07.

The valence of the prime did significantly influence valence ratings but it made no difference whether the prime referred to the self or others. Therefore, the hypothesis that valence ratings would be intensified with congruent self-referential primes was not supported.

## Task 3: Ratings of Self-Esteem

Having made valence ratings for the faces, participants were then asked to indicate how being confronted with each face made them feel about themselves. It was hypothesized that on trials where there was a negative self-referential prime followed by a face that was then rated negatively, then the rating of the participant’s self-esteem would be lower than in the other prime-face pairings. Similarly, it was expected that a face that was rated positively, preceded by positive self-referential information would result in higher self-esteem than other prime-face pairings.

### Results

A prime (5) x emotional configuration (5) repeated measures ANOVA was conducted on the self-esteem responses. There was a significant main effect of prime, *F*(1.84, 33.09) = 17.88, *MSE* = .20, *p* < .001, *η*_*p*_*²* = .5. Bonferroni contrasts indicated that negative self-referential primes led to significantly more negative self-esteem (*M* = 3.27, SE = .07) than negative non-self-referential primes (*M* = 3.07. SE = .04, *p* = .002), neutral (*M* = 2.95, SE = .04, *p* = .001), positive self-referential (*M* = 2.98, SE = .04, *p* = .005) and positive non-self-referential primes (*M* = 2.98, SE = .03, *p* < .001). Negative non-self-referential primes also made participants feel significantly more negative about themselves than neutral (*p* = .03) or positive non-self-referential primes (*p* = .004). No other contrasts were significant (*p* > .2). Therefore, negative primes appear to be more influential than positive primes to influence self-esteem overall.

There was also a significant main effect of emotional configuration on the self-esteem ratings, *F(*1.35, 24.34) = 39.04, *MSE* = 1.51 *p* < .001, *η*_*p*_*²* = .68, with Bonferroni contrasts showing that sad faces (*M* = 3.47, SE = .08) led to significantly more negative self-esteem than neutral faces (*M* = 3.26, SE = .04, *p* = .001), happy faces (*M* = 2.37, SE = .09, *p* < .001) or sad eyes/happy mouth faces (*M* = 2.83, SE = .07, *p* = .001), but not than happy eye/sad mouth faces (*M* = 3.43, SE = .08, *p* > .95).

Negatively categorized happy eyes/sad mouth faces also led to significantly more negative self-esteem than neutral faces (*p* = .002), happy faces (*p* < .001) and sad eyes/happy mouth faces (*p* = .001). Happy faces led to significantly more positive self-esteem than neutral faces (*p* < .001), as did sad eye/happy mouth faces (*p* = .002). Whilst increasingly positively perceived faces increased positive self-esteem, faces with negative mouths were equally influential in increasing negative self-esteem regardless of the expression of the eyes.

**Fig 4:**
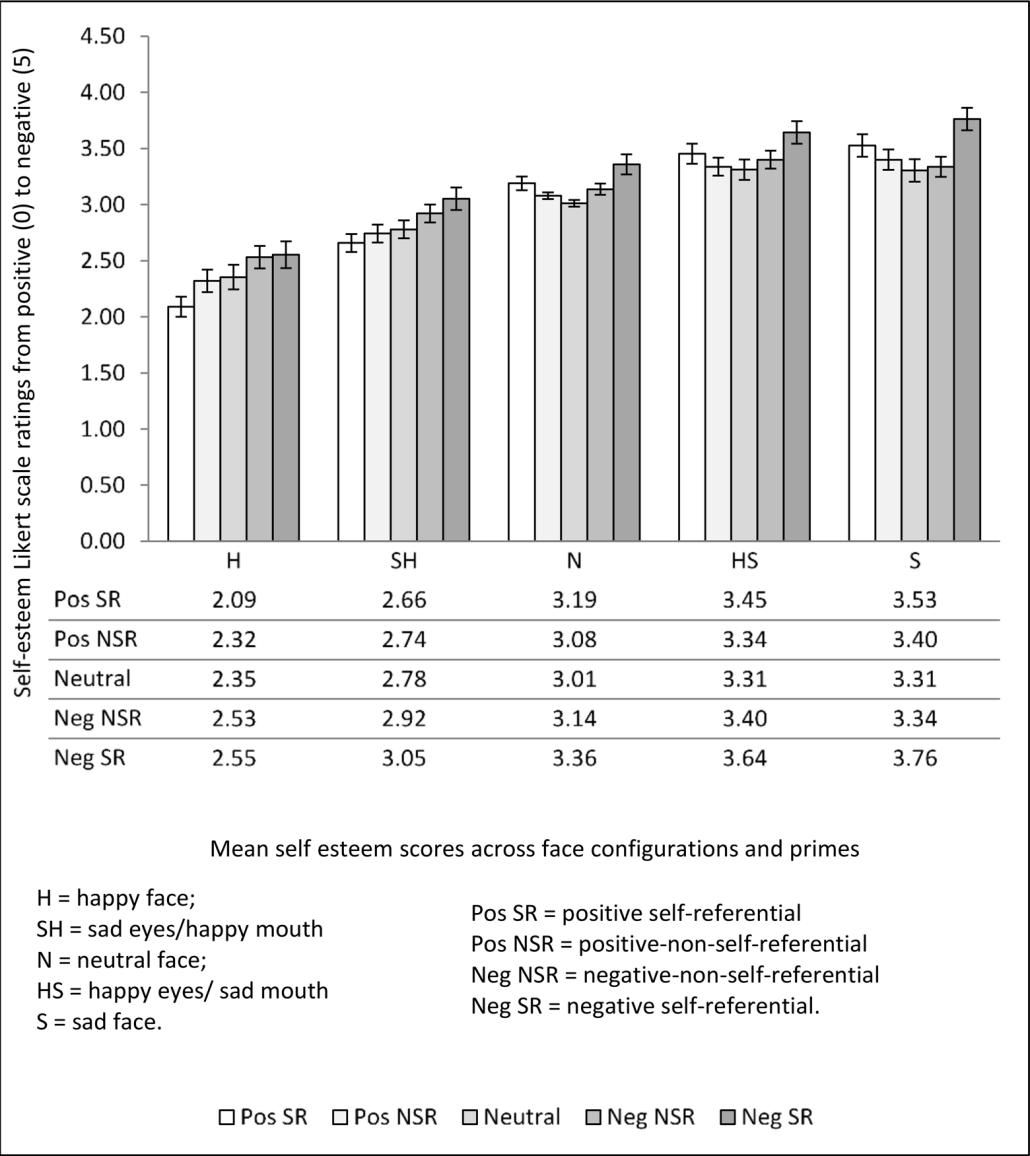
Means and standard errors for self-esteem scores across face configurations for each prime type.

The configuration x prime interaction was also significant, *F(*6.61, 118.94) = 6.22, *MSE* = .03, *p* < .001, *η*_*p*_*²* = .26. Means, standard deviations and *p* values for Bonferroni post hoc contrasts are illustrated in Fig 4 and Table 1. Faces with happy mouths made participants feel better about themselves if preceded by a positive prime. Positive self-referential primes in combination with an obviously happy face increased self-esteem most, and the presence of sad eyes significantly lowered positive self-esteem compared to when both the eyes and mouth was happy. Thus, positive self-esteem in the context of face processing requires the individual to pay attention to a positive face before their positive expectations will make them feel good about themselves. Yet a negative feature even when it is out of context has some influence in reducing positive self-esteem.

**Table 1:** Bonferroni post hoc p values for differences in self-esteem scores across prime types for each emotional configuration.

	H	SH	N	HS	S
Pos SR v Neg SR	.01	.07	.11	.04	.07
Pos NSR v Neg SR	.25	*<* .01	.22	.01	< .01
Neutral v Neg SR	.5	.01	.02	< .01	.001
Neg NSR v Neg SR	>.95	.38	.12	.03	< .01
Neg NSR v Pos SR	.001	.14	>.95	*>*.95	.03
Neutral v Pos SR	.001	.95	.09	.15	.13
Pos NSR v Pos SR	.03	>.95	.10	.23	.55

Note: SR = self-referential, NSR = non-self-referential.

As predicted the congruence of prime statements and perceived emotion from facial expressions intensified self-esteem.

## Discussion

The primary aims of the study were to investigate the influence of positive or negative self or non-self-relevant linguistic primes on attentional, cognitive and affective responses to emotional faces, with attentional allocation to faces assessed through eye-movement analysis. A language prime paradigm was used to mimic the types of thoughts that an individual may have when approaching a social situation. In line with Groner et al [54] and Itier and Batty [47] higher proportions of fixations and dwell times were made to the eyes in general. The primes influenced how positively or negatively faces were perceived, and the impact of the face on self-esteem was intensified with a congruent self-referential prime. However, despite congruency effects at a cognitive level, the primes had no effect on guiding eye movements which were solely guided by the features of the emotional configurations. The cognitive effects may to some extent reflect the LPP activity found in the study conducted by Diéguez-Risco et al [35] in terms of elaborative processing of congruency effects. The N170 congruency effects in the aforementioned study may facilitate speed of processing but the current study was not designed to measure this. Happy mouths appeared to be the most visually salient feature overall in terms of attracting attention and happy eyes the least salient. Ratings of the emotion of the face were based on the emotion of the mouth (in line with Malcolm et al [50]) despite minimal attention to mouths on the whole versus eyes. However, features that occupied the most attention influenced ratings.

It is possible that the overall preference for fixating on the eye region may represent an attempt for individuals to gauge the mental state or cognitions of another person in a social situation. Yet it more likely reflected the greater salience of two combined features. Indeed in composite faces with happy mouths, sad eyes being more salient than happy eyes did draw some attention away from happy mouths, and valence ratings were relatively less positive. Overwhelmingly, the eye movement data suggested that fixations were guided by salience alone as the primes had no influence on fixations or dwell time. These results are inconsistent with the findings of Aviezer et al [15], which suggest that context guided fixations to eye and mouths in a pattern consistent with the expectation. However, the present study differed from that of Aviezer et al in a number of significant ways. The principal difference was of course the nature of the priming information employed, which in the Aviezer study was emotional body context. It is distinctly possible that such a prime led to some degree of prepotent saccadic behaviour in their participants, which was not the case in the present study where primes both differed in nature and were presented *prior* to the face displays.

The eye movement and ratings results reflect the findings of Calvo and Nummenmaa [57], who demonstrated that judgements were made after just one fixation, with an average duration of less than 200 ms. The speed of this judgement may explain why the primes had no effect on guiding fixations when deciding the valence of the face, although it should be borne in mind that in the present study our participants were not asked to make an explicit judgment whilst viewing the face, only after offset. The results also suggest that valence ratings appear to be less susceptible to self-reference whilst self-esteem ratings were more self-focused.

Whilst self-referential primes influenced self-esteem, salience of the facial features also had a significant impact. Negative eyes in a face rated as positive reduced positive self-esteem. The eye movement data suggests that sad eyes were more salient than happy eyes, but less salient than a happy mouth, so in a face that had been rated as being positive based on the more salient happy mouth, even limited attention to sad eyes was enough to negatively impact self-esteem. Yet this was exacerbated by the expectation of a negative face. Self-referential primes that were consistent with the valence ratings of the face led to more extreme self-esteem ratings. It is possible that this process is connected to neural areas within the superior temporal sulcus which Mobbs et al [33] have suggested, as a region of multisensory integration, is involved in the integration of context and salient features.

Thus, primes influenced the self-esteem of the viewer despite having no effect on eye movements although there was no difference between self-referential and non-self-referential primes on valence ratings. This suggests that self-referential thoughts during face processing are a reactive rather than an instructive process in relation to emotional face processing. How one feels about one’s self, or how one perceives a person to be thinking about them, does not appear to influence the way in which a person will rate a facial expression. Rather, the act of paying attention to (albeit briefly) and subsequently rating the emotion on a face can interact with preconceived ideas about the underlying cognition of the expression to affect self-esteem. ERP data from Herbert et al [20] suggests that that personal relevance of emotional words may affect semantic processing stages. Coupled with the results of the current study, it may be the case that enhanced implicit processing for self-referential word primes during attentional capture feeds into cognitions about the self rather than influencing the visual attentional processes that can be detected in eye-movement analysis.

Conclusions from this study must be interpreted in light of some limitations. For example, the valence ratings were made before the self-esteem ratings on each trail. This could potentially have resulted in an order effect where the self-esteem responses were based on the decision that has already been made about the valence of the face rather than the direct effect of the face on self-esteem. However, this seems less likely given the results which indicated that although faces with sad eyes and happy mouths were rated on average positively, they were associated with less positive self-esteem when a negative expectation preceded them.

A further potential confound is that the noses used in the composite faces were all neutral and it is possible that a face split in half whereby the nose incorporated elements of each emotion would have resulted in different outcomes. Additionally, whilst the faces could have been vertically misaligned, as was conducted in one condition by Calder et al [32], our stimuli were designed to look as natural as possible in whole face configurations. It would not have been possible to achieve this as effectively by this method. Our manipulation afforded us the opportunity to directly examine the influence of primed expectations relating to the face on attentional allocation to features compatible with that expectation in whole faces. Alternatively, the corresponding emotion from the eyes could have been used, and separately for the mouth, but since the study already involved a lengthy eye-tracking experiment this was deemed to be impractical.

It is not possible from these experiments to ascertain the relevance of the findings in terms of psychiatric disorders, which was quite out with the scope of the study, but if similar results were found then it suggests that attentional biases could potentially be due to more basic processing differences rather than being guided by expectations. This may have implications for research in areas where socio-cognitive-behavioral models (e.g. Rapee and Heimberg [42]) suggest that expectations can prime vigilant attention in more socially anxious individuals. It may in fact be the case that the attentional patterns operate relatively independently of social cognitions and that faces are first categorized before the relevance to the self is processed. To gain more knowledge on this, it would be useful to replicate the study using clinical samples and composite faces with different negative emotions as well as a variety of self-relevant behavioral measures.

The behavioral measures used in this study were taken after the face had disappeared from the screen but it may also be useful to capture this information in real time whilst viewing the face in order to measure reaction time to decision making for more personally relevant measures. It may also be beneficial to pair self-relevant behavioral responses with EEG measures and eye-tracking. Finally, it would be informative to investigate the effect of negative expectations on initial orientation to features in congruent and incongruent face composites with clinical samples.

Is it possible for linguistic primes to directly influence saccadic behaviour? We would argue that our preliminary data cannot preclude the possibility, although of course our exploratory factorial study employed a modest sample of 19 participants, and awaits further research. However, our data indicates that in the present context the attentional draw of eyes is strong. However, we have only began to explore a paradigm that consists of a plethora of methodological considerations relating to exposure duration, participant instructions and participant decisions to name a few. To provide one example, we opted to randomize the presentation of primes, rather than blocking them by category. Methodologically it could be argued that this placed an unrealistic load on participants, who found themselves having to continuously switch between contexts across trials. Prime sentences were mixed across blocks rather than being block presented so that we could capture the effect of the types of thoughts that people may generate quickly on approaching a social situation without being prepared. In effect these would be transitory expectations which block priming could not adequately capture, as with block priming the participant may habituate to the nature of the prime. We would argue that such a task more closely reflects real life experience.

Given the potential implications of priming in future applications, particularly with regard to individuals with dysfunctional facial biases, we would argue that it is an area worthy of future study.

In conclusion, language as a prime appears to have no direct effect on attention when viewing emotional faces. Rather, attention appears to be dependent on visual salience alone but both salience and primes influence the effect on the viewer at a cognitive level. Linguistic primes appear to have a role to play in perceiving the intensity of the emotion during elaborative processing stages involving social cognition. This may have implications for facilitating social confidence since it appears that a combination of manipulating self-relevant expectations and attention towards non-threatening facial features may be more effective than attention training or cognitive reappraisal alone.
